# A Novel Assay for Detection of Methicillin-Resistant *Staphylococcus aureus* Directly From Clinical Samples

**DOI:** 10.3389/fmicb.2020.01295

**Published:** 2020-06-18

**Authors:** Jo-Ann McClure, John M. Conly, Osahon Obasuyi, Linda Ward, Alejandra Ugarte-Torres, Thomas Louie, Kunyan Zhang

**Affiliations:** ^1^Centre for Antimicrobial Resistance, Alberta Health Services/Alberta Precision Laboratories/University of Calgary, Calgary, AB, Canada; ^2^Department of Pathology and Laboratory Medicine, University of Calgary, Calgary, AB, Canada; ^3^Department of Microbiology, Immunology and Infectious Diseases, University of Calgary, Calgary, AB, Canada; ^4^Department of Medicine, University of Calgary, Calgary, AB, Canada; ^5^The Calvin, Phoebe and Joan Snyder Institute for Chronic Diseases, University of Calgary, Calgary, AB, Canada; ^6^Alberta Health Services, Calgary, AB, Canada

**Keywords:** *Staphylococcus aureus*, methicillin-resistant *Staphylococcus aureus* (MRSA), staphylococcal cassette chromosome *mec* (SCC*mec*), coagulase negative staphylococci (CoNS), MRSA rapid detection assay, molecular diagnosis

## Abstract

The timely detection of Methicillin-resistant *Staphylococcus aureus* (MRSA) is crucial for antimicrobial therapy and a key factor to limit the hospital spread of MRSA. Currently available commercial MRSA detection assays target the 3′ end of the *orfX* gene and the right extremity of Staphylococcal Cassette Chromosome *mec* (SCC*mec*). These assays suffer from both false positive due to SCC-like elements that lack *mecA* and false negative results due to the inability to detect new or variant SCC*mec* cassettes with the existing primers. We developed a novel MRSA detection scheme, designed to circumvent issues present in the existing commercial assays. Our assay demonstrated specificity and accuracy, capable of detecting prototypic strains of SCC*mec* types I-XIII [C(t) values ranged 8.58–26.29]. Previous false positive isolates (*N* = 19) by Xpert MRSA nasal assay were accurately classified with our assay. Further validation with 218 randomly selected clinical isolates (73 MRSA, 75 MSSA, 43 MR-CoNS, and 27 MS-CoNS) confirmed its feasibility and practicality. Testing assay performance with 88 direct clinical swabs from 33 patients showed that the assay was 96.6% in agreement with clinical culture results. Our novel MRSA detection assay targets both the *S. aureus* specific sequence and the *mecA*/*mecC* genes simultaneously to overcome the false positive and false negative deficits of currently available commercial assays. The results validate our assay and confirmed its feasibility and practicality. The assay is not affected by SCC*mec* types and only needs modification if new *mec* homologs emerge and establishes a new platform for other emerging SCC*mec* types.

## Introduction

Methicillin-resistant *Staphylococcus aureus* (MRSA) strains emerged shortly after clinical methicillin use (Barber, 1961; Jevons, 1961) through acquisition of the methicillin resistance gene *mecA*, which is carried on a mobile genetic element, staphylococcal cassette chromosome *mec* (SCC*mec*) (International Working Group on the Classification of Staphylococcal Cassette Chromosome Elements [IWG-SCC], 2009). The SCC*mec* element is characterized by terminal repeat regions, two main genetic components (the *ccr* and the *mec* gene complexes), and three joining regions (J1–J3) which are located between the *ccr* and the *mec* gene complexes and repeat regions (Ito et al., 2001). Currently 13 SCC*mec* types have been described (I-XIII), based on the nature of the *ccr* and the *mec* gene complexes that they carry, and further classified into subtypes according to differences in their J region DNA (Lakhundi and Zhang, 2018). The J regions can be shared between SCC*mec* types, or can be unique to a specific type. Strains of MRSA have spread and become established as major nosocomial pathogens worldwide (Crossley et al., 1979; Panlilio et al., 1992; Voss et al., 1994; Ayliffe, 1997; Fluit et al., 2001), and more recently have emerged as a major cause of community-acquired infections (Vandenesch et al., 2003; Lindsay and Holdenm, 2004). Although 1st generation cephalosporins (e.g., cefazolin and cephalexin) and isoxazolyl penicillins (oxacillin, cloxacillin) are still the agents of choice for treatment of methicillin-susceptible *S. aureus* (MSSA) infection, MRSA is resistant to most β-lactam agents, including cephalosporins (with the exception of ceftibiprole and ceftaroline) and carbapenems. The timely detection of MRSA is crucial for optimal management of this pathogen and for limiting the nosocomial spread of this organism.

Early detection is complicated by the fact that in clinical samples *S. aureus* (SA) is often mixed with less pathogenic coagulase-negative staphylococci (CoNS), both of which can harbor *mecA*. Conventional methods for the detection of MRSA in clinical microbiology laboratories depend on growth of the organism with selective media, which is time consuming and requires 2–3 days. Nucleic acid amplification methods are also used to discriminate MRSA from MSSA and CoNS, however, traditional genetic identification methods require PCR amplification of a pure bacterial culture.

Currently, two FDA-approved commercially available PCR based assays are widely used to detect MRSA directly from clinical samples, including IDI-MRSA/GeneOhm MRSA (BD Diagnostics, Franklin Lakes, NJ, United States) and GeneXpert MRSA (Cepheid, Sunnyvale, CA, United States). Both assays are similar, using quantitative real-time PCR-based methods targeting the 3′ end of the *orfX* gene in SA, in conjunction with the J3 region at the right extremity of staphylococcal cassette chromosome *mec* (SCC*mec*), but not targeting *mecA* directly. However, both assays are problematic in that they produce false negative and false positive results. False negatives arise from the inability to detect new, variant and non-typeable SCC*mec* cassettes with the existing primers, and false positives from the presence of SCC-like elements that do not contain *mecA*, which are incorrectly amplified (Desjardins et al., 2006; Malhotra-Kumar et al., 2008; Kelley et al., 2009; Arbefeville et al., 2011; Stamper et al., 2011). Moreover, any emerging variant SCC*mec* new types will not be detected. In recent years these assays have been updated to improve sensitivities and specificities, and to include the *mecA/C* genes, such as with the BD Max MRSA XT, Xpert gen3, and BD Max StaphSR assays (Ellem et al., 2015; Lepainteur et al., 2015; Silbert et al., 2015, 2017; Becker et al., 2016; Mendes et al., 2016). They still, however, rely on detection at the SCC*mec*-*orfX* junction and could suffer from the lack of ability to detect emerging SCC*mec* variants.

Here we present a novel scheme for the direct detection of MRSA from clinical samples, which is designed to circumvent the false positive and negative issues of the currently available commercial assays. This novel 2-step assay uses a small number of primers that simultaneously target both the *mecA/C* and SA specific *orfX* genes and are able to accurately detect all MRSA tested to date, including SCC*mec* control types I-XIII.

## Materials and Methods

### Bacterial Strains and Culture Conditions

Bacterial strains were grown overnight on Tryptic soy agar plates at 37°C. Control strains, including SCC*mec* I (NCTC10442), type II (N315), type III_Hg_ (85/2082), type IIIA (JCSC290), type IVa (CA05), type IVb (8/6-3P), type IVc (MR108), type IVd (JCSC4469), IVg (JCSC6673), IVh (JCSC6674), IVi (JCSC 6668), IVj (JCSC6670), type V (WIS [WBG8318]-JCSC3624), VII (JCSC6082), IX (JCSC6943), X (JCSC6945), and XI (LGA251) were obtained from K. Hiramatsu and T. Ito at the Juntendo University in Tokyo, Japan. SCC*mec* IIA (AR14/0298), IIB (AR05/0.1345), IIC (AR14/2188), IID (AR13/3635.2), IIE (AR13/3330.2), IVE (AR43/3330.1), and IVF (AR43/3636.1) were obtained from D. Coleman at the University of Dublin, Ireland. SCC*mec* VI (HDE288) was obtained from H. de Lencastre at The Rockefeller University, New York, United States. SCC*mec* XIII was obtained from M. Stegger at the Statens Serum Institut in Copenhagen, Denmark. SCC*mec* IIb (05MS-150) and VIII (C10682) were recovered from patients in our local hospitals or clinics. Strains Iowa 1-20 were obtained from S. Richter, University of Iowa Health Care, Iowa, United States (Arbefeville et al., 2011).

### DNA Extraction

DNA was extracted by rapid boiling method as previously described (Zhang et al., 2004, 2008). Alternatively, purified DNA was extracted using the QIAamp DNA mini kit (Qiagen Inc., Toronto, ON, Canada) as per the recommended protocol, with elution in 50 μl of sterile distilled water.

### Primer Design

Primers and probes for the MRSA detection real-time PCR assay were designed following comprehensive analysis of the *orfX* and *mecA* genes, in both CoNS and SA. Primers and their sequences are listed in Table 1.

**TABLE 1:** Primers and probes used in the first and second round PCR reactions*^*a*^*.

Primer/Probe	Sequence*^b^*	Target	References
**Round 1 long range PCR reaction**			
SA-3e	biotin-TTACACCAGACTTGCACTCGGATTGGCCCAAGAATTGAACC	*orfX*	This study
Umec-F	GGCCACGCGTCGACTAGTACCCAGGTTCAACYCAAAAAATATTAAC	*mecA*	This study
Umec-FX	GGCCACGCGTCGACTAGTACTTGGAACGATGCCTATCTCATATGC	Reversed *mecA* of SCC*mec* X	This study
**Round 2 real-time PCR reaction 1 (*orfX* reaction)**			
Arb3	TTACACCAGACTTGCACTCGG	Right side tail of SA-3e	This study
SA-4	CACTTGTTCAATTAACACAACC	*orfX*	This study
*SA-HEX-1	5′-/5HEX/CGGATCAAA/ZEN/CGGCCTGCACAAG/3IABkFQ/-3′	*orfX*	This study
**Round 2 real-time PCR reaction 2 (*mecA* reaction)**			
Arb2	GGCCACGCGTCGACTAGTAC	Left side tail of Umec-F/Umec-FX	Caetano-Anolles, 1993
Umec-R	ATGTTRTCTGATGATTCTATTGCTTG	*mecA*	This study
Umec-RX	GGAAGTTAGATTGGGATCATAGCG	Reversed *mecA* of SCC*mec* X	This study
*mec-FAM-2	5′-/56-FAM/AGGGTTGGC/ZEN/AAAAAGATGCATCATGGG/3IABkFQ/-3′	*mecA*	This study
*mec-FAM-3	5′-/56-FAM/AAGGTTGGC/ZEN/AAAAAGATAAATCTTGGG/3IABkFQ/-3′	*mecA*	This study
*mec-FAM-4	5′-/56-FAM/CGTGGTAAA/ZEN/ATTTTAGACCGAAACAATGTG/3IABkFQ/-3′	Reversed *mecA* of SCC*mec* X	This study

*^*a*^*Primers used in each reaction are listed, with their corresponding sequences and gene/sequence target. *^*b*^*The tail sequences were underlined. *Indicates probes.

### Long Range PCR Conditions

Round one LR-PCR used primers SA-3e, Umec-F and Umec-FX (Table 1). DNA template was derived from either crude boiled extracts, or from Qiagen kit extraction as described. One μl of template DNA was added to 24 μl of reaction mixture containing 0.2 mM of each primer, 1X LA PCR buffer II (TaKaRa Bio inc), 0.4 mM of each dNTP (TaKaRa Bio inc), and 1 unit of LA Taq HS (TaKaRa Bio inc). Amplification was performed in a 2720 thermal cycler (Applied Biosystems, Foster City, CA, United States) using 15 cycles of 98°C for 10 s, 58°C for 15 s, and 68°C for 20 min, followed by holding at 4°C.

### Magnetic Bead Capture and PCR Product Washing

Ten μl of 10 mg/ml streptavidin coated magnetic beads (Dynabeads M-270 streptavidin; Invitrogen) was used for each LR-PCR reaction. Beads were pooled in a 1.5 ml microcentrifuge tube and washed twice in 1 ml of 1X binding and washing buffer containing 5 mM Tris-HCL, pH7.5, 0.5 mM EDTA, and 1 M NaCl, with separation from the buffer accomplished with a DynaMag-2 (Invitrogen, Oslo, Norway). Following the washes, beads were re-suspended in 20 μl of 2X binding and washing buffer (10 mM Tris-HCL, pH7.5, 1 mM EDTA, 2 M NaCl) per reaction, and 20 μl of suspension added to each LR-PCR tube. Tubes were incubated at room temperature for 45 min, with gentle mixing by inversion every 5 min. Following binding, magnetic beads were washed two times with 100 μl of 1X binding and washing buffer, with mixing and separation accomplished using a DynaMag-96 Side (Invitrogen, Oslo, Norway). The samples were re-suspended in 100 μl of 10 mM Tris buffer, then 50 μl transferred to each of 2 real-time PCR tubes. Using the magnet, buffer was removed from the tubes, leaving the DNA bound beads as template for the second round real-time PCR.

### Round 2 Real-Time PCR Conditions

Round 2 real-time PCR was set up as 2 reactions for each sample. Detection of the SA specific *orfX* gene was accomplished with the first reaction, using primers Arb3 and SA-4. The 5′ PrimeTime qPCR 5′ nuclease probe, SA-HEX-1, was used for PCR product detection (Integrated DNA Technologies, Skokie, IL, United States) (Table 1). Detection of the *mecA/C* gene was accomplished with the second reaction, using primers Arb2 along with primers Umec-R and Umec-RX. The 5′ PrimeTime qPCR 5′ nuclease probes mec-FAM-2, mec-FAM-3 and mec-FAM-4 were used for PCR product detection in this reaction (Integrated DNA technologies, Skokie, IL, United States) (Table 1). The reactions were made using 1 mM of each of the appropriate primers, along with 0.25 mM of each corresponding probe and 1X PrimeTime gene expression master mix (Integrated DNA technologies, Skokie, Illinois), in a final volume of 10 μl. Ten μl of the *orfX* mix was added to one of the tubes containing washed beads, while 10 μl of the *mecA* mix was added to the second tube of washed beads. Both reactions were run simultaneously in the same real-time PCR thermocycler with the following conditions: an initial incubation at 95°C for 2 min was followed by 41 cycles of 95°C for 10 s and 65°C for 20 s, with at HEX read for the tubes containing the *orfX* mix, or a FAM read for tubes containing the *mecA* mix. Reactions were considered positive when the C(t) value was between 1-38, and negative if there was no C(t) value, or if it was greater than 38.

### Assay Sensitivity, Specificity and Validation

The ability of our MRSA detection assay to detect a large variety of SCC*mec* types was assessed using SCC*mec* control strains, including types I, II, IIA, IIB, IIC, IID, IIE, IIb, III_Hg_, IIIa, IVa, IVb, IVc, IVd, IVE, IVF, IVg, IVh, IVi, IVj, V, VI, VII, VIII, IX, X, XI, and XIII (Table 2). Assay cross-reactivity was determined using a collection of 40 non-MRSA, including 30 non-staphylococcal strains (Table 3). One μl of Qiagen purified DNA was used as template.

**TABLE 2:** The assay correctly detected SCC*mec* type prototypic strains.

SCC*mec* type	Strain (Accession number)	Approximate size of LR product (Kbp)	*orfX* detection with SA-HEX-1 C(t) Mean	*mecA* detection with mec-FAM-2/3/4 C(t) Mean	Result interpretation
I	NCTC10442 (AB033763)	6.5	13.45	16.09	+
II	N315 (D86934)	11.8	17.40	22.33	+
IIA	AR14/0298	CND	8.58	15.68	+
IIB	AR05/0.1345	CND	12.92	18.46	+
IIC	AR14/2188	CND	13.55	20.68	+
IID	AR13/3635.2	CND	14.61	15.40	+
IIE	AR13/3330.2 (AJ810120)	CND	13.26	15.67	+
IIb	05MS-150	CND	24.28	15.55	+
III_Hg_	85/2082 (AB037671)	42.3	16.33	26.29	+
IIIA	JCSC290	CND	14.93	22.27	+
IVa	CA05 (AB06372)	6.6	15.15	17.35	+
IVb	8/6-3P (AB063173)	6.6	13.68	15.77	+
IVc	MR108 (AB096217)	9.2	15.32	18.24	+
IVd	JCSC4469 (AB097677)	CND	14.26	14.55	+
IVE	AR43/3330.1 (AJ810121)	CND	14.10	16.02	+
IVF	AR43/3636.1	CND	12.45	15.36	+
IVg	M03-68 (DQ106887)	6.6	11.66	13.38	+
IVh	JCSC6674	CND	15.69	15.44	+
IVi	JCSC6668 (AB425823)	6.6	16.17	17.78	+
IVj	JCSC6670 (AB425824)	6.6	16.23	15.51	+
IVk^†^	45394F (GU122149)	32.6	NA	NA	NA
V	JCSC3624 (AB121219)	6.2	14.69	16.04	+
Vt^†^	JCSC7190 (AB512767)	14.3	NA	NA	NA
VI	HDE288 (AF411935)	6.3	16.24	19.98	+
VII	JCSC6082 (AB373032)	20.9	15.58	21.46	+
VIII	C10682 (FJ390057)	6.5	11.70	14.02	+
IX	JCSC6943 (AB505628)	23.8	19.04	25.46	+
X	JCSC6945 (AB505630)	6.1*	14.3	12.54	+
XI	LGA251 (FR821779)	2.3	14.45	12.89	+
XII^†^	BA01611 (KR187111)	39.2	NA	NA	NA
XIII	55-99-44 (MG674089)	25*	22.73	19.27	+
NTC			0	0	–

CND, could not determine since the full sequence is not available in NCBI. *^†^*NA, strain was not available to test. While SCC*mec* XII has also been described (Wu et al., 2015), it has not been possible to obtain the strain from the authors so we could not test it. * *mecA* gene is inserted into the chromosome in the opposite orientation. “+”: strain determined to be MRSA. “–”: Strain determined to be non-MRSA.

**TABLE 3:** Assay specificity in various strains of coagulase-negative staphylococci and non-staphylococcal bacteria.

Genus/species	Strain	*orfX* detection with SA-HEX-1	*mecA* detection with mec-FAM-2/3/4	Interpretation
**Staphylococci**				
*S. epidermidis*	ATCC 12333	–	+	MR, but not MRSA
*S. epidermidis*	ATCC 35984	–	+	MR, but not MRSA
*S. epidermidis*	ATCC 12228	–	–	Not MRSA
*S. haemolyticus*	ATCC 29970	–	–	Not MRSA
*S. saprophyticus*	ATCC 15305	–	–	Not MRSA
*S. sciuri*	ATCC 29060	–	–	Not MRSA
*S. simulans*	ATCC 27851	–	–	Not MRSA
*S. xylosus*	ATCC 29971	–	–	Not MRSA
**Non-staphylococci**				
*Acinetobacter baumannii*	ATCC 19606	–	–	Not MRSA
*Aeromonas caviae*	ATCC 15468	–	–	Not MRSA
*Alcaligenes faecalis* subsp. *faecalis*	ATCC 8750	–	–	Not MRSA
*Bacillus cereus*	ATCC 11778	–	–	Not MRSA
*Bordetella pertussis*	ATCC 9340	–	–	Not MRSA
*Citrobacter freundii*	ATCC 43864	–	–	Not MRSA
*Corynebacterium diphtheria*	ATCC 11913	–	–	Not MRSA
*Edwardsiella tarda*	ATCC 15947	–	–	Not MRSA
*Enterococcus durans*	ATCC 6056	–	–	Not MRSA
*Erysipelothrix rhusiopathiae*	ATCC 19414	–	–	Not MRSA
*Escherichia coli*	OP50	–	–	Not MRSA
*Klebsiella pneumonia*	ATCC 35657	–	–	Not MRSA
*Kocuria rosea*	ATCC 186	–	–	Not MRSA
*Lactococcus lactis*	ATCC 11454	–	–	Not MRSA
*Leclercia adecarboxylata*	ATCC 23216	–	–	Not MRSA
*Legionella pneumophilia*	ATCC 33152	–	–	Not MRSA
*Listeria monocytogenes*	ATCC 19115	–	–	Not MRSA
*Macrococcus caseolyticus*	JCSC5402	–	–	Not MRSA
*Micrococcus luteus*	ATCC 4698	–	–	Not MRSA
*Moraxella catarrhalis*	ATCC 49143	–	–	Not MRSA
*Mycobacterium smegmatis*	ATCC 14468	–	–	Not MRSA
*Neisseria gonorrhoeae*	ATCC 31426	–	–	Not MRSA
*Oligella ureolytica*	ATCC 43534	–	–	Not MRSA
*Ralstonia pickettii*	ATCC 49129	–	–	Not MRSA
*Salmonella typhymurium*	ATCC 14028	–	–	Not MRSA
*Serratia marcescens*	ATCC 43862	–	–	Not MRSA
*Shigella sonnei*	ATCC 25931	–	–	Not MRSA
*Stenotrophomonas maltophilia*	ATCC 51331	–	–	Not MRSA
*Streptococcus pneumonia*	ATCC 49136	–	–	Not MRSA
*Streptococcus pyogenes*	ATCC 19615	–	–	Not MRSA
*Streptomyces griseus*	ATCC 10137	–	–	Not MRSA
*Vibrio parahaemolyticus*	ATCC 17802	–	–	Not MRSA

**^*a*^*MR, methicillin-resistant.*

To determine assay sensitivity, the correlation of 1.0 McFarland standard being equal to 3 × 10^8^CFU/ml was initially used. Control strains carrying SCC*mec* II, III, IVa, IX, X, and XI were suspended in saline to a concentration of 1.0 Mcfarland standard. Exact colony counts were further confirmed by plating dilutions of the suspension on TSA plates, and growing overnight at 37°C. One ml of suspension was pelleted and the DNA extracted following standard procedures using the QIAamp DNA mini kit, and eluted with 50 μl of sdH_2_O. DNA concentration of the extract was determined with a Qubit 3 fluorometer using the Qubit dsDNA BR kit (Invitrogen). Ten fold serial dilution of the DNA were subsequently used to assess assay sensitivity.

The ability of the assay to correctly classify isolates falsely identified as MRSA by the Xpert MRSA nasal assay (Arbefeville et al., 2011) was determined. One μl of boiled DNA from 20 MSSA strains (Iowa 1–20) was tested. The assay was subsequently validated using a collection of strains that were obtained from random clinical samples that had been extensively characterized molecularly (Zhang et al., 2004, 2008). DNA was extracted using the boiling method and 1 μl used in the assay.

### Clinical Applicability Using Direct Patient Swabs

The ability of the assay to detect MRSA directly from patient samples was assessed with 88 swabs collected from hospitalized patients previously known to be MRSA positive or from a convenience sample of patients attending the sexually transmitted infection clinic in our health region. Duplicate swabs were collected from multiple sites on each patient appropriate to the clinical setting [nasal (N), throat (T), perianal-perineal (K), groin (G), Z-swab (Z), wound (W), axilla (A), or vaginal (V) swab]. One swab was subjected to routine clinical culture and identification, followed by genetic characterization of each isolate with a PCR multiplex assay capable of distinguishing CoNS from SA and methicillin-resistant vs. methicillin-susceptible isolates (Zhang et al., 2008). Isolates were also subjected to SCC*mec* typing (Zhang et al., 2012) as previously described. DNA was isolated from the second swab using the QIAmp DNA minikit (Qiagen Inc., Toronto, ON, Canada) following protocol D for isolation of genomic DNA from gram-positive bacteria, using 200 μg/ml of lysostaphin. The entire swab was deposited into the microcentrifuge tube for extraction purposes, and DNA was eluted from the column with 50 μl of sterile water. One μl of the eluent was used as the template in real-time PCR assay. When PCR was found to be positive and clinical culture negative, the swab used for clinical culture was placed into 30 ml of Tryptic soy broth and grown overnight at 37°C. Fifty μl of the overnight culture was transferred to a blood plate and subjected to clinical identification and molecular typing, as described above.

## Results

### A Novel Scheme for Direct MRSA Detection Designed to Overcome the Deficits of Commercially Available Assays

Currently available commercial MRSA detection assays rely on quantitative real-time PCR-based methods targeting the 3′ end of the *orfX* gene along with the right extremity of SCC*mec*, but not directly targeting the *mecA* gene. Both, however, suffer from the detection of false positives (due to SCC-like elements that lack *mecA*) and false negatives (due to the inability to detect new, variant or non-typeable SCC*mec* cassettes with the existing primers). We developed a novel MRSA detection assay scheme, designed to overcome issues present in the existing commercial assays.

**FIGURE 1:**
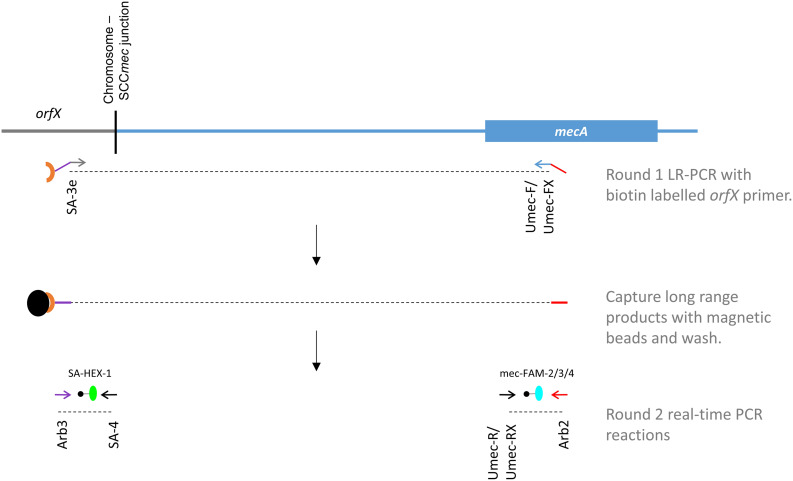
A new scheme for the MRSA directly clinical sample detection, designed to circumvent deficits in commercially available assays. Primers for the initial long range (LR) PCR are located in the *orfX* and *mecA* genes (green and blue arrows respectively) and contain tail sequences (indicated by purple and red lines) that are unique, and function as templates for Round 2 primers (indicated by purple and red arrows). Primer SA-3e is biotin labeled (brown crescent), allowing the LR-PCR product to be captured by streptavidin coated magnetic beads (black orb), and concentrated/purified. The Round 2 real-time PCR reactions are detected with probes specific to SA’s *orfX* (green orb) and *mecA* (blue orb).

Our novel assay is comprised of 2 PCR steps (Figure 1), including an initial long range (LR-) PCR reaction (Round 1), the product of which acts as the template for the second round real-time PCR reactions (Round 2). Forward and reverse primers for LR-PCR are located in the *orfX* and *mecA/C* genes, creating a PCR product that ranges in size from 2.3 to 42.3 kb (Tables 1, 2). By simultaneously targeting both of these regions, the assay was designed to eliminate templates that do not contain the *mecA* gene, as well as anything that is not SA. Primer Umec-F targets a conserved sequence in the *mecA*/*mecC* gene, present in all SCC*mec* types except types X and XIII. Since the orientation of *mecA* in SCC*mec* X and XIII is reversed compared to the other types, primer Umec-FX was included to effectively detect those types, as well as any future SCC*mec* types with the reversed *mecA* orientation. Primer SA-3e targets a region that is specific to the SA *orfX* gene and is universally found in all SA with available sequences. The target sequence differs significantly enough from that found in CoNS, thereby preventing amplification from CoNS *orfX* genes. Primer SA-3e also carries a 5′ biotin label, allowing the LR-PCR products to be captured with streptavidin coated magnetic beads, thereby concentrating them and purifying them of residual LR-PCR reaction components. Magnetic beads containing captured LR-PCR product from Round 1 were added directly to the round 2 real-time PCR reaction mixtures as template. All 3 LR-PCR primers were also designed with a 20 or 21 bp tail sequence on the 5′ end that do not match any bacterial genomes published thus far (Table 1, underlined sequence). These right (*orfX* side) and left (*mecA* side) tail sequences act as templates for round 2 PCR primers, eliminating the possibility of amplification directly from any contaminating chromosomal DNA in the Round 2 reactions.

The second round real-time PCR was designed as 2 separate reactions; one to detect the *orfX* gene, and the other to detect the *mecA* gene. For the *orfX* reaction, amplification primer SA-4 is specific to a region in the SA *orfX* gene, while primer Arb3 is specific to the right side- tail of primer SA-3e. Probe SA-HEX-1, also specific to a region in the SA *orfX* gene, was used to detect the PCR product specific for SA. For the *mecA* reaction, primer Arb2 is specific to the left side tail of UmecF/UmecFX, while primer UmecR is specific to the *mecA* gene in the typical orientation, and Umec-RX is specific to the *mecA* gene in the reversed orientation. Because of slight sequence variations in the *mecA* gene sequence, probes mec-FAM-2 and mec-FAM-3 were both needed to detect the PCR products, and mec-FAM-4 needed for detecting the product from SCC*mec* X and XIII.

Many layers of specificity were built into the assay by careful selection of the target sequences for the long-range primers, round 2 primers and probes. However, due to the erroneous nature of long-range PCR, whereby short PCR products can be generated from one correctly annealing and one mis-priming primer, we determined that it was essential to detect from both the *mecA* and *orfX* sides. While these mis-primed products are too few to detect on a traditional gel, they become amplified in the Round 2 reactions and create signals in one reaction or the other. In MSSA we noted that there was a positive signal from the *orfX* reaction, while in MR-CoNS we noted a positive signal from the *mecA* reaction. In each case, however, the signal from the other reaction was negative, allowing us to discriminate between these strains and true MRSA, which are the only ones positive for both the *mecA* and *orfX* reactions.

**FIGURE 2:**
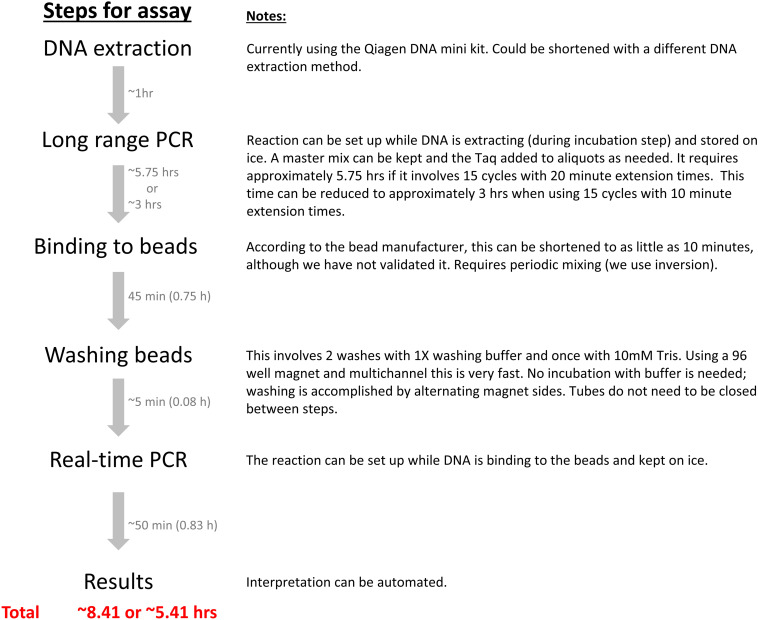
New MRSA detection assay timeline. The assay steps and approximate timelines are diagramed with the detailed explanation in the right side notes. Automation in a closed system could decrease assay time, minimize the numbers of manual procedures required, and reduce or eliminate the possibility of contamination.

A diagram of the assay steps and approximate timelines with the detailed explanation is outlined in Figure 2.

### New Assay Detecting SCC*mec* Types I-XIII

**FIGURE 3:**
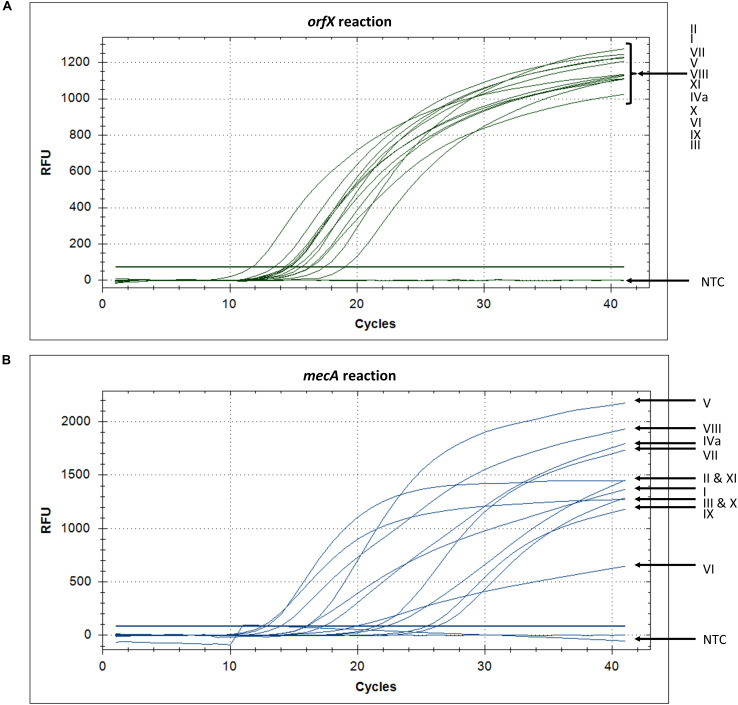
*orfX* and *mecA* reaction amplification curves show that SCC*mec* types I–XI are detected. **(A)** Detection of SCC*mec* types with the *orfX* reaction, using fluorophore HEX, shows that all types pass the threshold and have C(t) values between cycles 11 and 19, while the no template control (NTC) remains negative below the threshold. **(B)** Detection of SCC*mec* types with the *mecA* reaction, using fluorophore FAM, shows that all types pass the threshold and have C(t) values between cycles 13 and 27, while the no template control (NTC) remains negative below the threshold.

**FIGURE 4:**
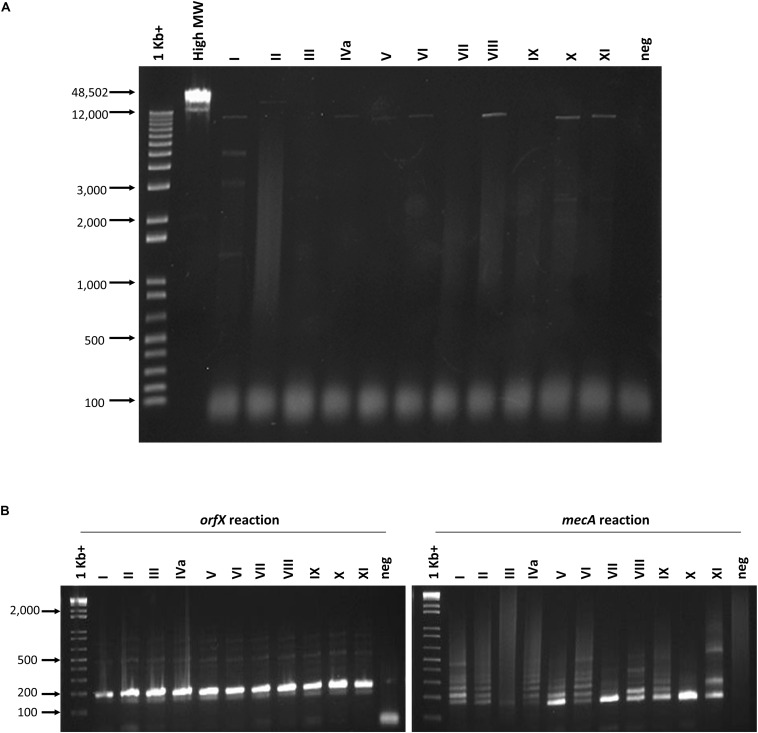
Agarose gel electrophoresis of round 1 and round 2 PCR reactions. **(A)** No PCR products corresponding to the correct sized product were seen following 10 cycles of long range amplification. Lanes from left to right correspond to the following samples: 1 Kb + marker (Invitrogen), High molecular weight marker (Invitrogen), SCC*mec* I–XI, no template negative control. **(B)** Round 2 *orfX* and *mecA* reactions show the correct sized amplicon in the *orfX* reaction, while most types produce weak and multiple bands in the *mecA* reaction. Lanes from left to right in each gel correspond to the following samples: 1 Kb + marker (Invitrogen), SCC*mec* I-XI, no template negative control.

The ability of the assay to detect a wide variety of SCC*mec* types was assessed with 28 types or subtypes, including SCC*mec* I-XIII. When the set of SCC*mec* types was tested, all 28 types and subtypes were found to be positive for both *orfX* and *mecA* reactions and, consequently, positively identified as MRSA with our assay (Table 2). Corresponding real-time PCR curves for each of the round 2 amplicons are shown in Figures 3A,B. C(t) values for the *orfX* reaction ranged from 8.58 (type IIA) to 24.28 (type IIb), while C(t) values for the *mecA* reaction ranged from 12.54 (type X) to 26.29 (type III_Hg_). During initial assay development, LR-PCR products from representative SCC*mec* types I-XI were run on a 0.7% agarose gel to visualize them (Figure 4A). Anticipated bands were not seen for any SCC*mec* type, however, with the small number of cycles being used, this result was not unexpected. Multiple bands were seen for some types (such as in type I), while in other types a smear was seen (such as type II and VII–XI). As with the LR-PCR products, second round real-time PCR reaction products were also run on a 2% agarose gel during assay development (Figure 4B). With the *orfX* reaction, the anticipated band was seen strongly at 196 bp for all representative SCC*mec* types, along with weaker bands that were larger in size. For the *mecA* reaction, multiple bands were seen for most SCC*mec* types, with the expected one at 213 bp being weak (or equivalent to the other ones) (data not shown). The notable exception was in SCC*mec* X, where the expected band at 179 bp was the only one visible. Despite the non-ideal results seen with these gels, the small number of full-length amplicons generated during LR-PCR were sufficient to act to act as templates for the second round reactions, with the product correctly detected by virtue of the probe specificity (Figure 3).

### Specificity of the Assay

Assay specificity was assessed using a panel of 40 non-MRSA, including 32 non-Staphylococci strains. In total there were 6 CoNS species (including 3 strains of *S. epidermidis*), and 31 strains from other diverse genera (including 2 species of *Streptococcus*) (Table 3). All of the isolates, including the 7 CoNS species, were negative with the *orfX* reaction, and all but 2 of the isolates were negative with the *mecA* reaction. This indicates that the assay does not cross react with other species and is very specific to MRSA. The noted exceptions were ATCC29971 (*S. xylosus*) and ATCC35984 (*S. epidermidis*), both of which are MR-CoNS and, consequently, positive for the *mecA* target.

### Sensitivity of the Assay

MRSA assay sensitivity will be impacted by the length of the long range PCR product (with longer products amplified with lower efficiency) as well as the efficiency of interaction of the specific primer pair in relation to the template DNA. As such, assay sensitivity was examined with 6 representative SCC*mec* types, covering a range of *orfX*-*mecA* interval sizes, both *mecA* and *mecC* genes, as well as the reversed *mecA* of type X. Under standard conditions, the limit of detection for the *orfX* reaction was 2.8–4.4 × 10^3^ CFU/PCR, and the limit of detection for the *mecA* reaction was 2.8–4.4 × 10^4^ CFU/PCR. As both targets need to be positive for a sample to be considered MRSA positive, the overall limit of detection for the assay is limited by the least sensitive reaction (the *mecA* reaction in most cases) (Table 4).

**TABLE 4:** Assay sensitivity in various representative SCC*mec* type prototypic strains.

Strain (SCC*mec* type)	Long range PCR size (Kb)	Limit of detection – *orfX* reaction (CFU/PCR)	Limit of detection – *mecA* reaction (CFU/PCR)	Overall limit of detection for assay (CFU/PCR)
N315 (II)	11.8	17	17	17
85/2082 (III_Hg_)	42.3	3.2 × 10^2^	3.2 × 10^4^	3.2 × 10^4^
CA05 (IVa)	6.6	72.4	7.2 × 10^2^	7.2 × 10^2^
JCSC6943 (IX)	23.8	4.4 × 10^3^	4.4 × 10^4^	4.4 × 10^4^
JCSC6945 (X)	6.1	16.4	16.4	16.4
LGA251 (XI)	2.3	2.8	2.8	2.8

### Previous False Positive Isolates Were Accurately Classified With Our MRSA Detection Assay

Strains Iowa 1–20, previously identified as false MRSA positives with the Xpert MRSA assay (Arbefeville et al., 2011), were tested with our MRSA detection assay and all 20 were found to be positive for the *orfX* reaction, while all but one were negative for the *mecA* reaction. Iowa-16 was positive for both the *orfX* and *mecA* reactions and classified as MRSA, which is in agreement with the Arbefeville study (Arbefeville et al., 2011).

### Assay Validation With Random Clinical Isolates

Validation of our assay was done using a collection of 218 randomly selected clinical isolates that had previously undergone extensive genetic characterization. Among them were 73 MRSA, 75 MSSA, 43 MR-CoNS, and 27 MS-CoNS isolates (Table 5). Of the 73 MRSA, 41 strains were previously determined to be SCC*mec* II, 10 were type III_Hg_, 3 were type IVa, 1 was type IVb and 18 carried an unknown SCC*mec* type. All 73 MRSA were positive in both the *mecA* and *orfX* reactions, with the *orfX* reaction having a lower C(t) than the *mecA* reaction [approximately 10 C(t) values lower (data not shown)]. All 75 MSSA had positive C(t) value for the *orfX* reaction but remained negative for the *mecA* reaction. Similarly, all 43 MR-CoNS had a positive C(t) value for the *mecA* reaction but remained negative for the *orfX* reaction. All 27 MS-CoNS were negative for both the *orfX* and *mecA* reactions. Table 5 summarizes results for the 218 clinical isolates, showing that the assay is capable of correctly identifying MRSA with 100% accuracy.

**TABLE 5:** Assay validation in 218 random clinical isolates.

Strain	Number of strains tested	*orfX* detection	*mecA* detection	Interpretation
				
		HEX probe	FAM probes	(% correct)
MRSA	73	+	+	MRSA (100%)
MSSA	75	+	–	SA but non-MRSA (100%)
MR-CoNS	43	–	+	MR but non-MRSA (100%)
MS-CoNS	27	–	–	Non-MRSA (100%)

*MR, methicillin-resistant; MS, methicillin-susceptible; CoNS, coagulase-negative Staphylococci.*

### Assay Applicability Determined With Direct Clinical Samples

A total of 42 samples were obtained from patients attending the sexually transmitted clinic, representing 14 patients with sampling from 3 different body sites for each patient. As seen in Table 6, none of the samples from the clinic were positive for MRSA using the real-time PCR assay, and no MRSA was isolated by routine clinical culture, meaning there was 100% agreement between the two methods. An additional 46 samples were obtained from hospital inpatients previously known to be MRSA positive, representing 19 patients with 2–3 body sites sampled for each patient. As shown in Table 7, there was a high degree of concordance between the real-time PCR assay results and clinical culture results. By clinical culture and PCR testing 23 and 25 patients were found to be MRSA positive, respectively, with 95.5% (84/88) agreement from the swabs. The PCR assay was able to detect low and high levels of MRSA on the swabs, ranging from 2 to several 100 colonies present on the plate. The results for a total of four swabs differed between the two assays. One swab was PCR negative, but 5–10 MRSA colonies were detected on the culture plate. One swab was PCR positive but plate culture negative, however, following overnight incubation of the swab in Tryptic soy broth (TSB), MRSA was detected in culture. Finally, two swabs were PCR positive but culture negative, and no MRSA was detected even after overnight incubation of the swabs in TSB broth. SCC*mec* typing of the MRSA that were isolated by clinical culture revealed that the majority of them (20/27) belonged to SCC*mec* IVa, 1/27 to SCC*mec* IVc, 2/27 to SCC*mec* II and 2/27 to SCC*mec* V. Two could not be determined because they were not isolated on plate culture, leaving no strain to type.

**TABLE 6:** Assay applicability assessed with swabs from random patients attending an outpatient clinic.

		Clinical Culture results						
		
Patient	Swab	MRSA	MSSA	MR-CoNS	MS-CoNS	Summary	Real-time PCR result	Assay agreement
1	N	–	–	–	+	No MRSA	–	√
	T	–	+	–	–	No MRSA	–	√
	K	–	–	+	+	No MRSA	–	√
2	N	–	+	–	+	No MRSA	–	√
	T	–	–	–	+	No MRSA	–	√
	K	–	–	+	+	No MRSA	–	√
3	N	–	+	+	–	No MRSA	–	√
	T	–	–	–	–	No MRSA	–	√
	V	–	+	–	+	No MRSA	–	√
4	N	–	+	–	+	No MRSA	–	√
	T	–	–	–	–	No MRSA	–	√
	K	–	–	–	+	No MRSA	–	√
5	N	–	+	–	+	No MRSA	–	√
	T	–	–	–	–	No MRSA	–	√
	K	–	–	–	+	No MRSA	–	√
6	N	–	+	–	+	No MRSA	–	√
	T	–	–	–	–	No MRSA	–	√
	K	–	–	+	+	No MRSA	–	√
7	N	–	–	–	+	No MRSA	–	√
	T	–	+	–	–	No MRSA	–	√
	K	–	–	–	+	No MRSA	–	√
8	N	–	+	–	+	No MRSA	–	√
	T	–	–	–	+	No MRSA	–	√
	K	–	–	–	+	No MRSA	–	√
9	N	–	–	–	+	No MRSA	–	√
	T	–	–	–	+	No MRSA	–	√
	K	–	–	–	+	No MRSA	–	√
10	N	–	+	–	+	No MRSA	–	√
	T	–	+	–	–	No MRSA	–	√
	K	–	–	–	+	No MRSA	–	√
11	N	–	–	–	+	No MRSA	–	√
	T	–	–	–	–	No MRSA	–	√
	G	–	–	–	+	No MRSA	–	√
12	G	–	–	+	–	No MRSA	–	√
	V	–	–	+	+	No MRSA	–	√
	T	–	–	–	–	No MRSA	–	√
13	N	–	–	+	+	No MRSA	–	√
	T	–	–	–	–	No MRSA	–	√
	G	–	–	+	+	No MRSA	–	√
14	N	–	+	–	+	No MRSA	–	√
	T	–	–	–	–	No MRSA	–	√
	G	–	–	–	+	No MRSA	–	√

*N, nasal; T, throat; K, perianal-perineal; V, vaginal; G, groin; + positive result (strain type was present or PCR was positive); −, negative result (strain type was not present or PCR was negative);, clinical culture and PCR assay agree.*

**TABLE 7:** Assay applicability assessed with swabs from hospital inpatients previously known to be MRSA positive.

		Clinical culture results								
		
Patient	Swab	MRSA	MSSA	MR-CoNS	MS-CoNS	# MRSA colonies	Summary	Real-time PCR result	Assay agreement	SCC*mec* type
15	N	+	–	+	–	◆◆	MRSA	+		IVa
	G	+	–	+	–	2	MRSA	+	√	IVa
16	N	–	–	+	+		No MRSA	–	√	
	Z	–	–	+	–		No MRSA	–	√	
17	N	–*	–	–	+	0	No MRSA*	+	√*	IVa
	G	–	–	+	+		No MRSA	–	√	
18	N	+	–	+	–	◆◆◆	MRSA	+	√	II
	G	+	–	+	+	◆	MRSA	–	√	II
19	N	–	–	+	–		No MRSA	–	√	
	G	–	–	+	–	0	No MRSA	+	×	Unk
	K	–	–	–	+		No MRSA	–	√	
20	N	+	–	–	–	◆◆◆	MRSA	+	√	IVa
	G	–	–	+	+	0	No MRSA	+	×	Unk
21	N	+	–	+	–	2	MRSA	+	√	IVa
	W	+	–	–	–	◆◆◆	MRSA	+	√	IVa
22	N	+	–	+	–	2	MRSA	+	√	IVc
	G	–	–	+	–		No MRSA	–	√	
	W	–	–	+	–		No MRSA	–	√	
23	N	+	–	+	–	◆◆	MRSA	+	√	IVa
	G	–	–	+	+		No MRSA	–	√	
24	N	+	–	+	–	◆	MRSA	+	√	IVa
	G	–	–	+	+		No MRSA	–	√	
25	N	+	–	–	–	◆◆	MRSA	+	√	IVa
	G	+	–	+	–	◆◆◆	MRSA	+	√	IVa
	K	+	–	+	–	◆◆◆	MRSA	+	√	IVa
26	N	+	–	+	–	◆◆	MRSA	+	√	IVa
	G	–	–	+	+		No MRSA	–	√	
	W	+	–	–	–	◆	MRSA	+	√	IVa
27	N	–	–	+	–		No MRSA	–	√	
	G	+	–	+	–	◆◆◆	MRSA	+	√	IVa
	W	+	–	+	–	◆◆◆	MRSA	+	√	IVa
28	N	+	–	–	+	◆	MRSA	+	√	IVa
	G	–	+	–	+		No MRSA	–	√	IVa
29	N	+	–	–	–	◆◆◆	MRSA	+	√	V
	R	+	–	+	–	◆◆◆	MRSA	+	√	V
30	N	–	–	+	+		No MRSA	–	√	
	G	–	–	+	–		No MRSA	–	√	
31	N	–	–	+	+		No MRSA	–	√	
	G	–	–	+	–		No MRSA	–	√	
	W	+	–	+	–	◆◆◆	MRSA	+	√	IVa
32	N	+	–	+	–	◆◆◆	MRSA	+	√	IVa
	G	–	–	+	–		No MRSA	–	√	
	W	+	–	+	–	◆◆	MRSA	+	√	IVa
33	N	–	–	+	–		No MRSA	–	√	
	G	–	–	+	–		No MRSA	–	√	
	A	–	–	+	–		No MRSA	–	√	

N, nasal; K, perianal-perineal; G, groin; Z, z-swab; W, wound; +, positive result (strain type was present or PCR was positive); −, negative result (strain type was not present or PCR was negative); ◆, 5–10 colonies; ◆◆, 11–100 colonies; ◆◆◆, 101–1000 colonies; √, clinical culture and PCR assay agree; ×, clinical culture and PCR assay do not agree; –* and No MRSA*, not found on plate but recovered after overnight incubation in TSB; √*, did not agree with initial plate culture, but agreed after recovery in TSB; unk, unknown because colonies not isolated.

## Discussion

Molecular detection methods have been developed but are hampered by the fact that clinical samples can be mixed, containing combinations of MSSA, MRSA, and MS- or MR-CoNS. Detection of the *mecA* gene does not necessarily indicate the presence of MRSA, as MR-CoNS could also be the source of the gene.

Two FDA-approved commercially available PCR based assays that have been described and are widely used to detect MRSA directly from clinical samples, include the IDI-MRSA/GeneOhm MRSA (BD Diagnostics) and GeneXpert MRSA (Cepheid). In the IDI-MRSA assay, there is a SA specific primer (Xsau325) in the *orfX* gene, immediately upstream of *attB*, along with a SA specific probes which gives species specificity. The other five SCC*mec* specific primers (mecii574, meciii519, meciv511, mecv492, mecvii512) are in the J3 region of SCC*mec*, and are used along with Xsau325 to amplify the junction region of MRSA (Huletsky et al., 2004). The GeneXpert MRSA assay is similarly designed, with primers spanning the chromosome *orfX*-SCC*mec* junction, with amplification and detection occurring in separate chambers of single-use disposable cartridge. Multiple studies, however, have found that both assays are problematic in that they produce false negatives from their inability to detect variant or new SCC*mec* types, and false positives from their detection of SCC-like elements that do not contain *mecA* primers (Desjardins et al., 2006; Malhotra-Kumar et al., 2008; Kelley et al., 2009; Arbefeville et al., 2011; Stamper et al., 2011). A study by Stamper et al. found that the GeneOhm MRSA assay had a sensitivity and specificity of 89 and 91.7% respectively, with false positives resulting from retained segments of the right extremity-junction sequences in non-*mecA* containing SCC-like elements (Stamper et al., 2011). Likewise, a study by Arbefeville et al. (2011) found that the rate of false positives for the Xpert MRSA assay was 7.7%, due to *mecA* dropout strains with remnants of SCC*mec* cassettes. Both assays are also unable to detect the newly emerging *variant SCCmec* types.

In 2015, the Xpert MRSA was updated to include targets for the *mecA* and *mecC* genes, in conjuction with the existing SCC*mec*-*orfX* junction (Becker et al., 2016). The new assay, GeneXpert MRSA Gen 3, was also updated to detect SCC*mec* VI–XI, on top of the I–V that it previously detected. A study by Lepainteur et al. (2015) detecting MRSA directly from nasal swabs showed that the assay had a sensitivity of 95.7% and a specificity of 100%. The BD Max MRSA XT (extended detection technology) kit was also introduced to detect *mecC* as well as *mecA* and, in the same study by Lepainteur, was shown to have a sensitivity and specificity of 87.5 and 97.1% respectively (Lepainteur et al., 2015). BD also released the BD Max StaphSR assay, which is a multiplex assay targeting the SCC*mec*-*orfX* junction, *nuc* and *mecA/C* genes, with a sensitivity of 99.1–100% and specificity of 100% when detecting from blood culture (Ellem et al., 2015). Further evaluation of the BD Max Staph SR and Max MRSA XT assays showed sensitivities of 94.3–99.1% and specificities of 97.7–99.8% (Silbert et al., 2015, 2017; Mendes et al., 2016). These assays, however, all target the SCC*mec*-*orfX* junction, and could be limited in their ability to detect newly emerging SCC*mec* types and variants.

Another FDA-approved assay, the cobas^®^ vivoDX MRSA test was announced in December 2019. The assay is a bioluminescence-based method that uses bacteriophage technology to detect MRSA in nasal swabs in as little as 5 h. While limited information is currently available about the assay (no publications available at present), they do report in media reports that the assay has a false negative rate of approximately 10%, and a false positive rate of 1.4%.

With the goal of improving point of care detection, we developed a new real-time PCR assay for the detection of MRSA in clinical samples that circumvents the major problems encountered with commercially available assays. Our assay simultaneously targets both the chromosomal *orfX* region and the *mecA* and has the advantage of only needing a small number of primers to detect a large range of SCC*mec* types. Because of the highly homologous nature of both the *orfX* and *mecA* genes, these limited primers are capable of detecting all SCC*mec* types tested to date, including SCC*mec* XI, which carries the new *mecC* homolog as well as SCC*mec* X and XIII, with *mecA* in the reversed orientation.

With the assay, we were able to correctly detect 28 SCC*mec* types and subtypes, ranging from SCC*mec* I to XIII. The high degree of assay specificity was further demonstrated by testing 40 non-MRSA isolates including 32 non-Staphylococcal species, and 218 clinical isolates that had previously undergone extensive genetic testing.

Applicability of the assay was assessed using clinical swabs from inpatients previously known to be MRSA positive, as well as from random patient samples from a clinic in our health region. Results indicate that the assay has a high level of sensitivity and accuracy. The one culture positive-PCR negative result could represent a false negative because the MRSA strain carried SCC*mec* II, which has a larger *orfX-mecA* size. However, the type II control strain had a low experimental limit of detection of 17 cells/reaction. It’s also possible that the discrepancy and false negative resulted from sampling error. The finding of 3 PCR positive and culture negative swabs suggests that the sensitivity of the real-time PCR assay is superior to that of culture, although as mentioned, sampling of the duplicate swabs could potentially account for the discrepancies, particularly if low levels of bacteria were present, illustrated by the one broth enriched positive culture. The differences could also be due to the presence of non-viable MRSA on the swabs. All three patients were on antibiotic therapy prior to the samples being collected. Assuming that the three swabs were in fact positive for MRSA, the real-time PCR assay correctly identified 87/88 (98.9%) of the swabs, while clinical culture correctly identified 85/88 (96.6%). An interesting observation was that the real-time PCR assay was able to detect MRSA with higher sensitivity than the experimental limits of detection suggested. Fourteen of the 25 swabs that were positive for MRSA had ≤100 colonies on the culture plates, with 9 of those having ≤ 10.

This novel MRSA detection assay has successfully overcome the issue of false negatives from new or novel SCC*mec* types, and false positives from non-*mecA* containing SCC-like elements (Desjardins et al., 2006; Malhotra-Kumar et al., 2008; Kelley et al., 2009; Arbefeville et al., 2011; Stamper et al., 2011). False negatives are eliminated due to the universal nature of the targets and primers chosen for the long-range PCR. By amplifying from these universal regions, rather than targeting sequences specific to each SCC*mec* type, this assay has been shown to detect the majority of SCC*mec* types and subtypes described to date (types IVk and XII were not available) and should, in theory, be effective in detecting newly discovered types. In fact, of the 73 MRSA clinical samples that were used to validate the assay, 18 of them were untypeable using previously described SCC*mec* typing assays (McClure et al., 2010; Zhang et al., 2012). Despite carrying an unknown SCC*mec* type, the assay was able to accurately classify them as MRSA. The second deficit of previously described MRSA detection assays was the issue of false positives, but with our design, and the simultaneous targeting of both *mecA* and *orfX* during the long-range PCR, these false positives have been eliminated. While the Arbefeville et al. (2011) study did not have a clear explanation for the false reactivity in the Xpert MRSA assay, they postulated that it could be due to the presence of SCC*mec*-*orfX* right extremity components. Screening these isolates with our assay correctly classified 19 of the strains as MSSA, and one as MRSA. SCC-like elements present in CoNS are also negative for both targets with our assay, eliminating this potential source of false positives. Of the 27 MS-CoNS clinical isolates that were tested, 3 of them were determined to have a non-*mecA* SCC element in previous studies (data not shown), and all remained negative for both targets with this assay. ATCC12228 is also described as carrying a non-*mecA* SCC-like element, known as SCC_pbp–__4_ (Mongkolrattanothai et al., 2004), and was negative for both targets with our assay.

In its current form, our novel assay does have some limitations. At present, 15 cycles of long-range PCR are used in the first round of amplification, which require 5.5 h to run. When factoring in sample preparation, handling time, magnetic bead capture/washing and the second round of PCR, the overall assay requires approximately 8–9 h (Figure 2). While this time frame is longer than the rapid turn-around times available with the 2 FDA approved assays, it is significantly shorter than the time required for traditional microbiology culture methods. Automation is an option for reducing assay time, as is decreasing the number of cycles and/or extension time of the long-range PCR. Initial attempts have been made to reduce the extension time to 10 min instead of 20 min and have shown promising results in both control strains and in clinical samples, which could potentially reduce the overall time from originally 8–9 h to about 6 h (Figure 2). Another limitation of the assay is the number of manual steps required, each of which increases the chances of contamination. Once again, automation in a closed system is a viable option for mitigating this problem. Currently, the assay clinical applicability was validated using the direct patient swab examples collected from different body sites including nasal, throat, perianal-perineal, groin, Z-swab, wound, axilla, and vaginal swabs. The possibility of application of the assay in other direct examples such as pus, wound, sputum and etc., should be assessed in future.

## Conclusion

Our MRSA detection real-time PCR assay has proven to be an effective method of identifying MRSA directly in clinical samples. Due to its design, in which both the *orfX* and *mecA/C* genes are simultaneously targeted, false positive and false negatives observed with other clinically approved point of care detection assays are eliminated. The assay can detect all SCC*mec* types tested to date and, in theory, should be effective at detecting most future ones that arise.

## Data Availability Statement

The original contributions presented in the study are included in the article/supplementary material, further inquiries can be directed to the corresponding author.

## Ethics Statement

The protocol for collection of the clinical swab samples from patients in this study was approved by the Conjoint Health Research Ethics Board, University of Calgary under Ethics ID: REB13-0219_MOD3 and REB16-0623_MOD1, on October 22, 2018 and April 9, 2019, respectively.

## Author Contributions

KZ conceived, designed, and supervised the work. J-AM, OO, and LW performed the experiments and analyzed the data. JC, AU-T, and TL collected and provided the clinical isolates. J-AM and KZ structured and drafted the manuscript. J-AM, JC, and KZ edited the manuscript. All authors reviewed and approved the manuscript.

## Conflict of Interest

The authors declare that the research was conducted in the absence of any commercial or financial relationships that could be construed as a potential conflict of interest.
